# Personality in Soccer: Investigation of the Five-Factor Model of Personality in High-Level Athletes

**DOI:** 10.3389/fspor.2022.896934

**Published:** 2022-05-26

**Authors:** Jan Spielmann, Adam Beavan, Jan Mayer

**Affiliations:** ^1^Department of Sports Sciences, Saarland University, Saarbrücken, Germany; ^2^TSG ResearchLab, Zuzenhausen, Germany

**Keywords:** big five, personality, NEO-FFI, reliability, item-analysis, high-level sports, soccer

## Abstract

**Background:**

In high-level sports, rapid screening and diagnostic instruments are necessary considering limited access that researchers have to these athletes. In the area of sport psychological diagnostics, the NEO-FFI is a promising tool to gain information about an athlete's personality traits. The current study investigated the NEO-FFI's scientific quality criteria and general application to elite-level soccer.

**Methods:**

Personality traits of 378 elite-level soccer athletes were assessed using the NEO-FFI. Analysis focused on internal consistency, factor structure and gender differences. Additionally, a second measurement with a 6-week interval was conducted with a sub-sample of 86 athletes to analyse test-retest reliability.

**Results:**

Overall, the results are in line with previous findings outside high-level sports. For the total sample, alpha-levels from 0.68 to 0.84 and intraclass correlation coefficients (ICC) for test-retest measures from 0.86 to 0.91 could be found. Item-level principal component analysis using both oblimin and oblique rotation showed better stability in neuroticism (N) and conscientiousness (C) than in extraversion (E), openness (O), and agreeableness (A). Gender differences could be found in values of internal consistency, ICC and NEO-FFI traits.

**Conclusion:**

The results of this study demonstrate good transferability of the NEO-FFI from settings outside high-level sports into this specific niche of sport psychological assessment. However, the same weaknesses of the applied instrument in general populations were also replicated in the sporting population.

## Introduction

In comparison to physiological approaches, psychological assessments in professional sports are rather seldom, particularly in soccer. Yet measuring the psychological aspects of athletes is rapidly gaining popularity and many of the well-established measurements from domains external to sport (i.e., cognitive and differential psychology) are now being applied in sporting domains. Examples of assessments that have already made this transition is the measurement of athletes' executive functions (Beavan et al., 2020), emotional behaviors (Schilaty et al., 2016), and of particular interest for this research, personality (Smith, 2008; Zhang et al., 2019). In the area of differential psychology, the classification of the Five Factor Model of Personality (FFM) is well-established (McCrae and Costa, 2008). The FFM assesses personality of an individual on five traits: Neuroticism (N), Extraversion (E), Openness (O), Agreeableness (A), and Conscientiousness (C); hence being commonly referred to as “the big five.” Despite some criticism of whether there are less (Eysenck and Eysenck, 1985; Gray, 1991; Zuckermann, 2005) or more than five dimensions (Paunonen and Jackson, 2000), the FFM is based on a general consensus in modern research and is widely accepted (O'Connor, 2002; de Moor et al., 2012; Allen et al., 2013; Bircher et al., 2017).

The NEO Personality Inventory (NEO-PI) and its subsequent revised version (NEO-PI-R) were developed as a measure of the FFM of personality (McCrae and Costa, 1985; Costa and McCrae, 1992). The NEO-PI-R contains 240 items that are grouped accordingly to one of the five personality dimensions. Despite the NEO-PI-R measuring narrower personality traits with more scales, the time commitment to complete the questionnaire is a limitation for many time-scarce populations, requiring up to 60 min to complete. In high performance settings where practitioners may be allocated limited time access to players, the use of the shorter version of the NEO-PI-R may be more appropriate (Egan et al., 2000). The NEO Five-Factor Inventory (NEO-FFI) is a shortened version of the NEO-PI-R, consisting of only 60 items that take between 10 and 15 min to complete (McCrae and Costa, 1987). It has demonstrated validity and utility in several different contexts and languages and is one of the most used instruments to assess the FFM (Egan et al., 2000; Zillig et al., 2002).

Reliability measures of the NEO-FFI has first been mentioned by Costa and McCrae (1992) in an English speaking sample, which has shown internal consistencies of 0.89 (N), 0.79 (E), 0.76 (O), 0.74 (A), and 0.84 (C) that were later supported (Holden and Fekken, 1994). Egan et al. (2000) similarly reported ranges between 0.72 (O) and 0.87 (N) on a large and diversified British cohort. Results from Eastern Europe and Iran also confirmed similar findings (Hrebíčková et al., 2002; Anisi, 2012). Of particular importance for the current study, the translation by Borkenau and Ostendorf (1993) found reliability indexes between 0.71 and 0.85 in a German population sample that were later on verified (Schmitz et al., 2001). Furthermore, test-retest reliability separated by a 2-week break has also been positively supported, ranging from 0.89 (N), 0.86 (E), 0.88 (O), 0.86 (A), and 0.90 (C) (Robins et al., 2001). Although the test-retest stability of the NEO-FFI is high, concerns for the factor structure of the NEO-FFI have been highlighted in the literature. Holden and Fekken (1994) conducted a factor analysis on the NEO-FFI using Canadian female students, reporting low loadings of ≥0.30 in 55 of 60 items. Various studies further reported some items not loading highly on their corresponding component when using factor analyses (Rolland et al., 1998; Egan et al., 2000; Aluja et al., 2005).

As a solution to the loading concerns, McCrae and Costa (2004) replaced 14 items of the original NEO-FFI with newer items taken from the NEO-PI-R in order to improve the instruments factor structure. The selection criteria were: (1) minimized effects of acquisition responding, (2) increased NEO-PI-R factor score correlation, and (3) diversification of item content in favor of underrepresented facets of remaining items of the scales. The newest version of the NEO-FFI showed correlations from 0.56 (O) to 0.62 (N) in self report adjective factors, 0.39 (C) to 0.53 (O) in NEO-PI factors in spouse and 0.34 (C) to 0.59 (O) in peer ratings (Costa and McCrae, 2008). Convergent correlation ranges from 0.34 to 0.62. The newest version of the NEO-FFI scales account for about 75–85% variance of the original NEO-PI factors for convergent criteria. However, remaining concerns about the loading were once more highlighted by Aluja et al. (2005) who compared the old and revised NEO-FFI version in a Swiss (*n* = 1,090) and Spanish (*n* = 1,006) sample with unsatisfying results, observing that 10 items did not fit into a perfect five-factor structure because of loadings being lower than 0.30.

Although the NEO-FFI has been described as a quick and effective inventory to measure the FFM and may be the preferable choice in time-scarce populations, the data on populations such as high-performance sport is insufficient. Practitioners and researchers alike would benefit from not having to rely on generalizations sourced from normative data outside sporting populations. Despite the wide range of studies using the NEO-FFI, there are less studies published that have specifically focused on physically active subjects (Allen et al., 2013; Wilson and Dishman, 2015; Piepiora, 2020). Moreover, no studies currently exist that target a large sample of athletes in elite-level populations, and specifically in soccer. Therefore, the current study aimed to examine the internal consistency, test-retest reliability, and trait- respective item-level analysis of the factor structure of the NEO-FFI on a sample of German national and international high-level soccer athletes. Additionally, a second aim was to analyse differences between male and female athletes in responding style and traits. It is hypothesized that the NEO-FFI will demonstrate to be reliable and suitable measure of the FFM in high-level athletes, consistent with the literature that has used this inventory in non-sporting populations.

## Method

### Participants

A total of 378 elite-level soccer athletes (210 male; 168 female) aged 16–37 (M = 19.86 years, SD = 4.38) participated in the study (Table 1). Inclusion criteria were German native speakers to prevent the dataset of biases sourced from aspects like misunderstanding or socialization in other cultural environments, age 16 and older, absence of self-reported suffer from a psychological disorder or any condition that would impair their results and being an athlete in one of the German professional league teams' club academy. In sum, all athletes were representing clubs of the professional soccer leagues within Germany at the time of the study, totalling 21 male and 18 female clubs. All athletes were team members ranging from the U17's to senior professionals. Altogether, 200 athletes (52.91%) have been or were currently part of a youth or adult National team of 15 different countries (mainly Germany, Switzerland, Austria).

**Table 1:** Participants' information for T1 and T2.

		**T1 (*n* = 378)**	**T2 (*n* = 87)**
Gender	Males	*n* = 210	*n* = 62
	Females	*n* = 168	*n* = 25
Clubs (pro league)	Male	1. Division *n* = 15 2. Division *n* = 3 3. Division *n* = 3 Total *n* = 21	1. Division *n* = 1
	Females	1. Division *n* = 12 2. Division *n* = 6 Total *n* = 18	1. Division *n* = 1
Age (years)	Mean (SD)	19.86 (4.38)	19.62 (3.96)
	Males	19.17 (4.52)	19.37 (4.22)
	Females	20.73 (4.05)	20.24 (3.21)
	Range	16–37	16–35
	Males	16–37	16–35
	Females	16–34	16–26
Team size	Males	U17 *n* = 75	U17 *n* = 15
		U19 *n* = 60	U19 *n* = 19
		U23 *n* = 37	U23 *n* = 17
		Pros *n* = 38	Pros *n* = 11
	Females	U17 *n* = 4	U20 *n* = 8
		U20 *n* = 76	Pros *n* = 17
		Pros *n* = 86	

*Participants' information for T1 and T2*.

*T1 represent the first measurement, T2 represent the second measurement 6 weeks post T1*.

### Personality Assessment

In order to determine the athletes' personality traits, the German NEO-FFI adaption by Borkenau and Ostendorf (2008) was used. The questionnaire consists of 60 items rated on a five-point Likert scale (strongly disagree, disagree, neutral, agree, strongly agree). It is a self-report measure that assesses the five personality dimensions: extraversion, neuroticism, openness, agreeableness, and conscientiousness. It was presented using an online survey (Microsoft Forms, Version 2020) in original form, order, and instruction.

### Procedure

Athletes involved in the study were tested *via* an online survey. The assessment had a standardized introduction and familiarization protocol, and a staff-member with sport psychological background could always be consulted. Before the participants started, they were informed that all results would remain anonymous, and participation was voluntary. Prior to the commencement of this study, informed consent from all athletes was received, and the Institutional Ethics Committee approved this study.

#### Testing Session 1 (T1)

The online survey of T1 was either forwarded by the team management of the different clubs or sent directly in terms of personal contact. In the case of one first division club (110 athletes, 71 males/39 females; M = 19.86 years, SD = 4.08), the questionnaire was part of a regular standardized, twice-yearly sports psychological performance diagnostics event recorded during pre-season. This team was chosen because of the test-retest reliability study as described below. Testing took ~10–15 min to read and complete the introduction, demographic information, and personality assessment.

#### Testing Session 2 (T2)

To assess test-retest reliability of the NEO-FFI, the first division club mentioned above were asked to complete the assessment again 6 weeks after T1. A window of +1 week was allowed for the retest. In total, 86 athletes (62 male, 24 female) aged between 16 and 35 (M = 19.62 years, SD = 3.96) participated at T2. The athletes completed the exact same testing as on T1.

### Data Analysis

The dataset was screened and checked for any kind of missing values and the relevant assumptions for parametric tests (i.e. outliers, independence, normality, sphericity, and homogeneity of variance) were measured and met in accordance with Tabachnick and Fidell (2014). Inspections of descriptive and graphical data analysis were executed to prove absence of outliers and normality distribution. Cronbach's alpha determined internal consistency. Test-retest reliability was calculated *via* an intraclass correlation coefficient (ICC) based on the dataset of 86 athletes using a single measure two-way mixed effect model with an absolute agreement type. Confidence intervals (95% CI) are reported. Gender differences were analyzed using an independent-sample *t*-tests for parametric and Mann-Whitney-*U* for non-parametric variables. In addition, Cohens' d effect-size was calculated. Exploratory factor analysis on trait- and item-level were based on principal component analysis with both oblique and orthogonal rotations.

## Results

### Internal Consistency

Cronbach alpha's internal consistency measures for T1 (Table 2) showed a range from 0.68 (O/A) to 0.84 (N) for all athletes. Interestingly, females showed higher reliability scores than males in all traits, exhibited as 0.71 (A) to 0.87 (N), 0.56 (A) to 0.80 (C), respectively.

**Table 2:** Descriptive NEO-FFI statistics (*n* = 378, plus gender separation, raw scores).

**Trait**	**All athletes (*****n*** **=** **378)**			**Males (*****n*** **=** **210)**			**Females (*****n*** **=** **168)**		
	**Mean**	**SD**	**Alpha**	**Mean**	**SD**	**Alpha**	**Mean**	**SD**	**Alpha**
N	15.60	6.96	0.84	13.80	6.08	0.78	17.84	7.35	0.87
E	31.06	5.05	0.69	31.06	4.72	0.64	31.07	5.45	0.75
O	24.92	5.37	0.68	23.73	4.59	0.57	26.42	5.90	0.73
A	32.22	4.80	0.68	30.67	4.14	0.56	34.15	4.87	0.71
C	36.60	5.70	0.83	37.04	5.13	0.80	36.04	6.32	0.86

### Test-Retest Reliability

One outlier could be detected in accordance with Tabachnick and Fidell (2014). To minimize its impact and to keep it as part of the population, a SQRT-transformation of the N-variables was conducted. For the 86 athletes who were retested, ICC test-retest reliability scores from 0.86 (E) to 0.91 (A) was observed (Table 3). In comparison, females (*n* = 24) had, except for C (ICC = 0.84 vs. 0.92 in males), higher scores ranging from 0.84 (C) to 0.94 (N) than males (*n* = 62) ranging from 0.84 (O) to 0.92 (C).

**Table 3:** Test-retest reliability of NEO-FFI traits, separated by gender.

**Trait**	**All athletes** **(*****n*** **=** **86)**					**Males** **(*****n*** **=** **62)**					**Females** **(*****n*** **=** **24)**				
	**T1**	**T2**	**ICC**	**CI**	**p**	**T1**	**T2**	**ICC**	**CI**	**p**	**T1**	**T2**	**ICC**	**CI**	**p**
	**Mean** **(SD)**	**Mean** **(SD)**		**95%** **(LL, UL)**		**Mean** **(SD)**	**Mean** **(SD)**		**95%** **(LL, UL)**		**Mean** **(SD)**	**Mean** **(SD)**		**95%** **(LL, UL)**	
N	13.28 (6.15)	13.60 (4.72)	0.91	0.86, 0.94	0.000	12.15 (5.26)	12.37 (6.11)	0.89	0.81, 0.87	0.000	16.21 (7.35)	16.79 (7.30)	0.94	0.86, 0.97	0.000
E	31.86 (4.96)	31.02 (4.19)	0.86	0.78, 0.91	0.000	31.98 (4.96)	31.19 (4.18)	0.86	0.76, 0.91	0.000	31.54 (5.05)	30.58 (4.26)	0.87	0.70, 0.94	0.000
O	24.35 (4.69)	24.72 (4.60)	0.88	0.81, 0.92	0.000	23.58 (4.39)	24.29 (4.40)	0.84	0.74, 0.91	0.000	26.33 (4.95)	25.83 (5.03)	0.93	0.85, 0.97	0.000
A	32.20 (4.27)	31.78 (4.36)	0.90	0.85, 0.94	0.000	31.21 (3.90)	30.89 (3.86)	0.89	0.81, 0.93	0.000	34.75 (4.20)	34.08 (4.79)	0.89	0.75, 0.95	0.000
C	37.86 (4.58)	37.88 (4.89)	0.89	0.84, 0.93	0.000	37.89 (4.56)	38.03 (4.81)	0.92	0.86, 0.95	0.000	37.79 (4.72)	37.50 (5.15)	0.84	0.63, 0.93	0.000

*T1 and T2 assessment distance of 6 weeks; ICC, intraclass correlation coefficient; CI, confidence interval with LL and UL representing the lower- and upper limit; p, significance value of ICC*.

### Intercorrelations Between NEO-FFI Trait Scores

**Figure 1:**
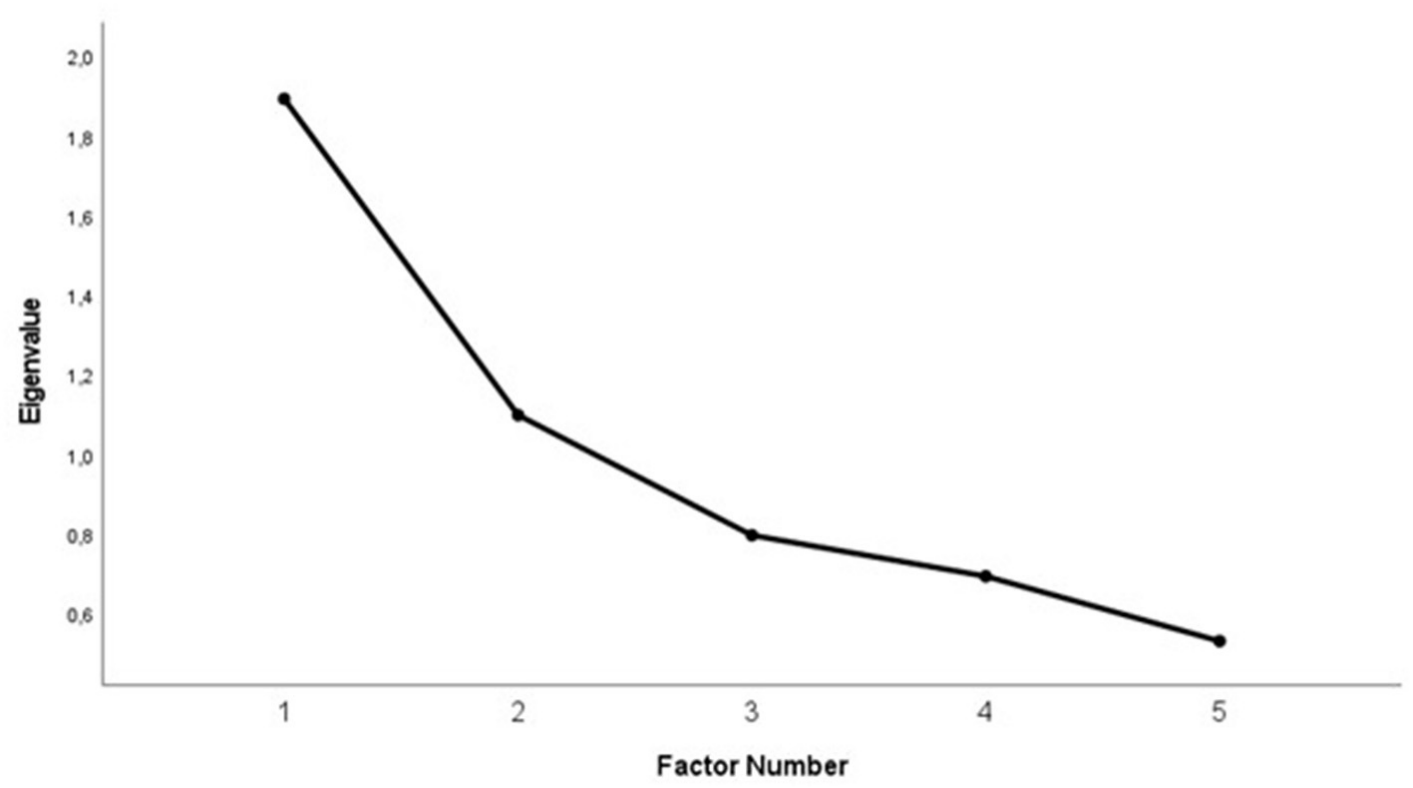
NEO-FFI trait-analysis. Component scree plot.

The NEO-FFI dimensions show various correlations (Table 4). Significant associations could be found for N with lower E and C, respectively, E with higher A and C. O was the only trait where no significant relations could be found. Principal component analysis with quartimax rotation of the orthogonal traits revealed a two-component solution (Figure 1) which converged in three iterations and explained 59.75% of the variance, with eigenvalues being 1.90 and 1.10. Table 5 shows results from component analysis. Component 1 had a high negative loading for N (−0.78) and high positive loadings for E (0.66), A (0.54), and C (0.67). Component 2 was defined by high loading of O (0.86) and lower loadings of E (0.42) and A (0.49).

**Table 4:** Intercorrelations between NEO-FFI trait scores (*n* = 378) on the upper right, *p*-values on the lower left, plus gender separation.

	^**a**^**All athletes (*****n*** **=** **378)**					^**b**^**Males (*****n*** **=** **210)**					^**c**^**Females (*****n*** **=** **168)**				
	**N**	**E**	**O**	**A**	**C**	**N**	**E**	**O**	**A**	**C**	**N**	**E**	**O**	**A**	**C**
N	–	−0.329***	0.046	−0.160**	−0.327***	–	−0.214**	0.017	−0.098	−0.403***	–	−0.486***	−0.046	−0.527***	−0.194*
E	*p =* 0.000	–	0.163**	0.384***	0.257***	*p* = 0.002	–	0.127	0.310***	0.281***	*p* = 0.000	–	0.179*	0.491***	0.232**
O	*p* = 0.373	*p* = 0.001	–	0.161**	0.043	*p* = 0.808	*p* = 0.067	–	0.033	0.094	*p* = 0.555	*p* = 0.020	–	0.081	0.016
A	*p* = 0.002	*p* = 0.000	*p* = 0.002	–	0.212***	*p* = 0.156	*p* = 0.000	*p* = 0.639	–	0.297***	*p* = 0.000	*p* = 0.000	*p* = 0.296	–	0.202**
C	*p* = 0.000	*p* = 0.000	*p* = 0.399	*p* = 0.000	–	*p* = 0.000	*p* = 0.000	*p* = 0.177	*p* = 0.000	–	*p* = 0.012	*p* = 0.002	*p* = 0.834	*p* = 0.009	–

*Two-tailed test*,

^***^p < 0.001,

^**^p < 0.01,

^*^p < 0.05.

*^a/c^Person's r for E and O; Spearman-Rho for N, A, and C*.

*^b^Person's r for E, O, and A; Spearman-Rho for N and C*.

**Table 5:** Component-analysis of the first two quartimax components extracted from analysis of the NEO-FFI traits (*n* = 378).

**Trait**	**Component 1**	**Component 2**
N	**−0.780**	0.137
E	**0.663**	**0.416**
O	**–**0.090	**0.863**
A	**0.537**	**0.494**
C	**0.666**	**–**0.134

*Loadings ≥0.30 are bold*.

### Item-Level-Analysis of the NEO-FFI

**Figure 2:**
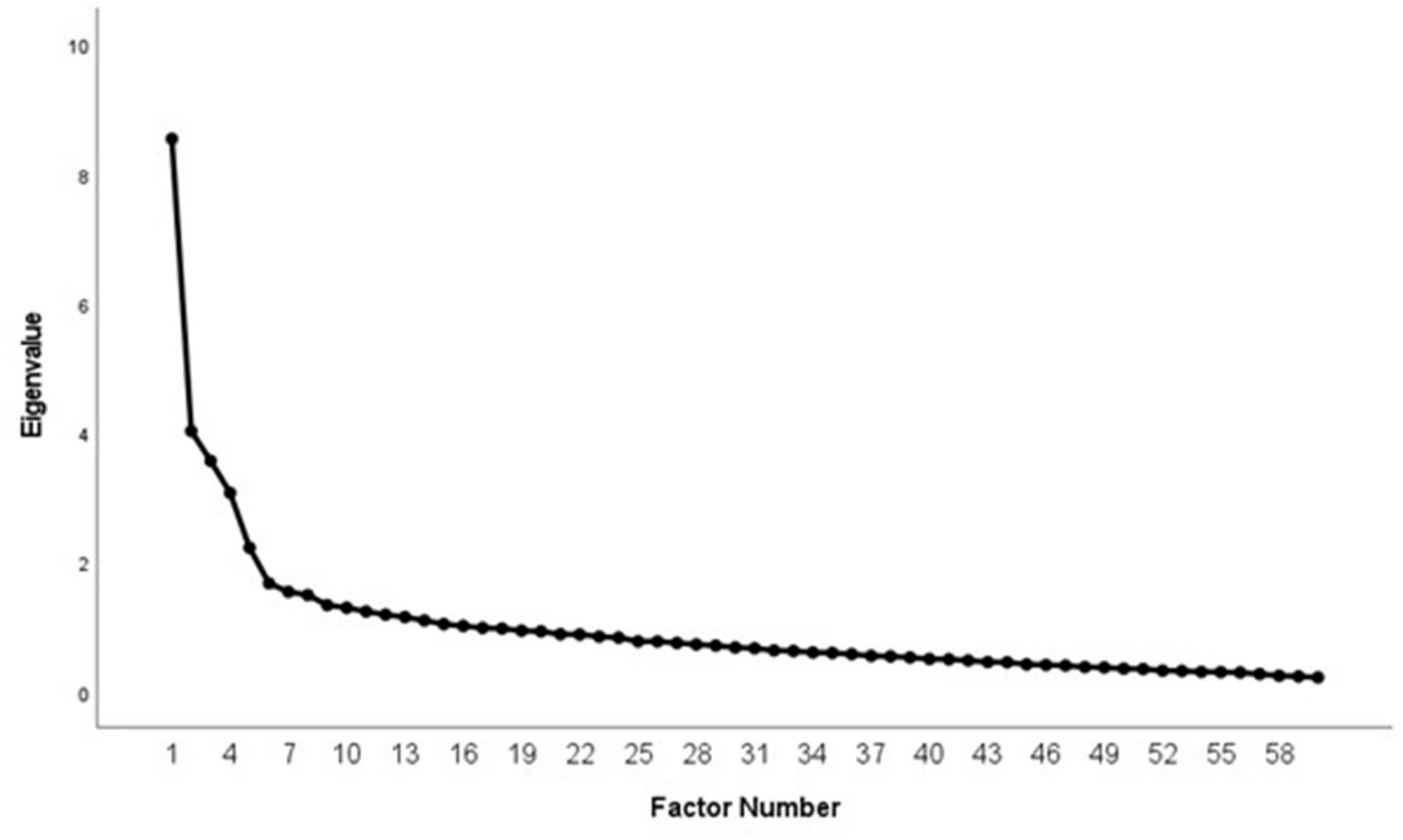
NEO-FFI item-analysis. Component scree plot.

For the analysis of sources of variance in the component solution, principal component analysis of the 60 items was conducted: 16 components with eigenvalues from 8.54 to 1.02 could be extracted. A total variance of 59.09% of the variance of the NEO-FFI could be explained. The components converged in 25 iterations in varimax rotation. Scree plot suggested a five-component solution with a 35.68% explanation of total variance as the main source of NEO-FFI (Figure 2).

#### Varimax Rotation of the NEO-FFI Items-5 Factor Solution

Table 6 displays loadings between the NEO-FFI Items and the first five orthogonal extracted components with varimax rotation. Items were re-ordered and labeled to improve readability.

**Table 6:** Component-analysis of the first five (orthogonal) varimax factors extracted from analysis of the NEO-FFI (*n* = 378).

	**Component**	**Component**	**Component**	**Component**	**Component**
	**1**	**2**	**3**	**4**	**5**
N1	−0.104	0.030	0.078	0.080	−0.051
N2	**0.666**	−0.060	−0.075	−0.072	−0.223
N3	**0.635**	−0.158	−0.082	0.060	0.040
N4	**−0.428**	−0.105	0.246	0.052	−0.092
N5	**0.689**	−0.049	−0.060	−0.013	0.008
N6	**0.746**	−0.116	−0.171	0.033	0.048
N7	**−0.573**	−0.092	−0.025	0.092	0.115
N8	**0.565**	−0.061	0.092	0.089	0.226
N9	**0.675**	−0.244	−0.141	−083	−0.076
N10	**−0.588**	0.103	0.149	−0.045	−0.081
N11	**0.747**	−0.238	0.004	−0.009	0.051
N12	**0.533**	−0.038	−0.091	0.079	−0.128
E1	−0.081	−0.083	**0.646**	−0.034	−0.147
E2	0.094	0.024	**0.590**	−0.016	−0.201
E3	**0.337**	−0.010	**−0.362**	−0.025	0.170
E4	−0.082	0.177	**0.683**	0.104	−0.045
E5	−0.108	−0.153	**0.483**	0.112	**0.347**
E6	0.198	0.100	−0.182	0.121	**0.382**
E7	−0.129	0.057	0.231	**0.402**	0.232
E8	**−0.362**	0.139	**0.655**	0.039	−0.102
E9	**0.328**	−0.043	**−0.528**	−0.134	0.117
E10	**0.361**	−0.276	0.132	0.182	0.096
E11	−0.184	0.274	**0.458**	0.127	0.078
E12	**0.329**	−0.205	−0.212	−0.023	0.183
O1	−0.128	0.248	0.082	−0.126	0.069
O2	−0.052	0.048	0.030	**−0.693**	0.125
O3	0.029	0.000	0.058	**0.583**	−0.019
O4	−0.007	0.067	0.103	−0.170	0.113
O5	−0.028	0.119	−0.073	**−0.530**	−0.039
O6	−0.087	0.144	0.107	**0.391**	−0.074
O7	−0.066	−0.198	−0.013	**−0.308**	0.183
O8	−0.028	0.156	0.173	−0.061	0.250
O9	0.007	−0.051	−0.093	**0.604**	0.097
O10	−0.041	−0.008	−0.031	**−0.664**	0.114
O11	−0.025	0.262	0.104	**0.524**	−0.017
O12	−0.021	−0.019	0.119	**0.622**	0.163
A1	−0.017	0.221	**0.437**	0.106	−0.141
A2	**0.345**	−0.199	−0.186	0.065	0.266
A3	0.008	−0.205	0.028	−0.076	**0.586**
A4	0.106	−0.049	0.090	0.120	**−0.400**
A5	0.299	0.009	−0.164	−0.032	**0.330**
A6	0.189	0.099	−0.017	−0.030	**0.516**
A7	−0.274	**0.303**	**0.348**	0.039	−0.041
A8	0.107	−0.171	**−0.396**	−0.125	**0.456**
A9	−0.026	0.036	−0.166	−0.008	**0.365**
A10	**0.335**	0.272	0.274	0.294	−0.272
A11	−0.010	−0.076	−0.135	−0.026	**0.466**
A12	0.071	−0.193	−0.102	0.140	**0.507**
C1	0.016	**0.619**	0.027	−0.059	0.057
C2	−0.131	**0.662**	−0.022	0.071	−0.054
C3	−0.094	**−0.379**	0.001	0.009	0.102
C4	−0.087	**0.615**	0.094	0.073	−0.058
C5	−0.278	**0.409**	0.222	0.012	**0.334**
C6	0.238	**−0.579**	−0.031	−0.010	−0.035
C7	**−0.398**	**0.373**	0.264	−0.046	**0.372**
C8	−0.047	**0.664**	0.103	0.086	−0.069
C9	0.107	**−0.695**	−0.037	−0.060	0.126
C10	−0.086	**0.688**	0.149	0.107	0.141
C11	0.217	**−0.653**	−0.016	0.074	0.002
C12	−0.016	**0.421**	0.143	0.059	**0.400**

*Loadings ≥0.30 in bold*.

Component 1 is clearly N, with all items except of N1 loading on the component. It also contains five items from the E component (E3, E8, E9, E10, E12), two from A (A2, A10) and one from C (C7). Component 2 represents unequivocally C, with all items loading on the dimension and one item of A (A7). Component 3 contains eight items of the trait E and three of A (A1, A7, A8). Component 4 contains nine items trait of O, but also E7. Component 5 contains eight items of A, with additional loadings of E (E5, E6) and C (C5, C7, C12).

Overall, the item-analysis showed quite separate traits for N, E, A, and C scales. O however is problematic, with three items which did not show any loadings ≥0.30 on any trait. In total, 47 of the 60 NEO-FFI Items represented unique items relating to specific traits. Four items (N1, O1, O4, O8) did not load on any of the first five factors (≥0.30). Seven items did not load on their intended dimension (E6, E7, E10, A1, A2, A7, A10).

#### Promax Rotation of the NEO-FFI Items-5 Factor Solution

Table 7 shows loadings between the NEO-FFI Items and the first five oblique extracted components with promax rotation. Items were re-ordered and labeled for improved reading purposes.

**Table 7:** Component-analysis of the first five (oblique) promax factors extracted from analysis of the NEO-FFI (*n* = 378).

	**Component**	**Component**	**Component**	**Component**	**Component**
	**1**	**2**	**3**	**4**	**5**
N1	−0.097	0.003	0.059	0.077	−0.052
N2	**0.691**	0.041	0.023	−0.098	−0.207
N3	**0.639**	−0.069	0.010	0.042	0.052
N4	**−0.428**	−0.213	0.220	0.050	−0.095
N5	**0.723**	0.053	0.038	−0.044	0.025
N6	**0.747**	0.006	−0.076	0.019	0.060
N7	**−0.640**	−0.176	−0.107	0.136	0.095
N8	**0.620**	−0.002	0.182	0.045	0.245
N9	**0.664**	−0.143	−0.024	−0.094	−0.063
N10	**−0.584**	0.006	0.075	−0.036	−0.090
N11	**0.773**	−0.150	0.140	−0.044	0.072
N12	**0.539**	0.045	−0.032	0.064	−0.119
E1	0.045	−0.202	**0.723**	−0.117	−0.120
E2	0.233	−0.055	**0.670**	−0.105	−170
E3	0.286	0.095	**−0.351**	0.005	0.164
E4	0.083	0.067	**0.719**	0.007	−0.016
E5	−0.029	−0.255	**0.535**	0.057	**0.364**
E6	0.189	0.162	−0.196	0.130	**0.378**
E7	−0.102	−0.002	0.190	**0.382**	0.233
E8	−0.227	−0.007	**0.659**	−0.037	−0.082
E9	0.241	0.087	**−0.522**	−0.081	0.104
E10	**0.367**	−0.269	0.212	0.158	0.108
E11	−0.061	0.192	**0.440**	0.062	0.095
E12	0.283	−0.138	−0.162	−0.005	0.181
O1	−0.071	0.238	0.060	−0.145	0.073
O2	0.013	0.057	0.103	**−0.715**	0.138
O3	0.000	−0.020	0.001	**0.588**	−0.025
O4	0.042	0.059	0.128	−0.192	0.122
O5	0.010	0.147	−0.038	**−0.537**	−0.033
O6	−0.079	0.114	0.044	**0.385**	−0.077
O7	−0.075	−0.209	0.037	**−0.302**	0.184
O8	0.044	0.138	0.181	−0.095	0.260
O9	−0.063	−0.053	−0.166	**0.633**	0.083
O10	0.000	0.008	0.039	**−0.675**	0.122
O11	−0.004	0.247	0.021	**0.512**	−0.019
O12	−0.041	−0.056	0.062	**0.622**	0.157
A1	0.101	0.162	**0.447**	0.038	−0.121
A2	**0.303**	−0.135	−0.140	0.079	0.265
A3	0.007	−0.216	0.073	−0.078	**0.588**
A4	0.107	−0.057	0.103	0.110	**−0.396**
A5	0.298	0.080	−0.135	−0.031	**0.332**
A6	0.231	0.138	0.006	−0.049	**0.522**
A7	−0.176	0.230	**0.309**	−0.007	−0.031
A8	0.023	−0.100	**−0.387**	−0.074	**0.441**
A9	−0.049	0.063	−0.188	0.012	**0.357**
A10	**0.434**	0.285	0.284	0.232	−0.253
A11	−0.037	−0.057	−0.135	−0.008	**0.460**
A12	0.028	−0.181	−0.091	0.158	**0.501**
C1	0.121	**0.661**	−0.033	−0.095	0.065
C2	−0.054	**0.690**	−0.130	0.053	−0.054
C3	−0.156	**−0.418**	0.033	0.032	0.096
C4	0.012	**0.627**	0.012	0.038	−0.052
C5	−0.180	**0.365**	0.163	−0.025	**0.340**
C6	0.163	**−0.580**	0.068	0.008	−0.035
C7	**−0.300**	**0.305**	0.205	−0.082	**0.377**
C8	0.063	**0.683**	0.019	0.045	−0.061
C9	0.011	**−0.719**	0.063	−0.031	0.123
C10	0.039	**0.697**	0.063	0.060	0.149
C11	0.128	**−** **0.666**	0.082	0.095	0.001
C12	0.085	**0.426**	0.106	0.018	**0.410**

*Loadings ≥0.30 in bold*.

Component 1 is clearly N, with all items except of N1 loading on the component. It also contains one items of E and C (E10, C7) and two of A (A2, A10). Component 2 represents unequivocally C, with all items loading on the dimension and no traits from other dimensions loading on this component. Component 3 contains eight items of the trait E and three of A (A1, A7, A8). Component 4 contains nine items of trait O, but also E7. Component 5 contains eight items of A, with additional loadings of E (E5, E6) and C (C5, C7, C12).

Overall, the item-analysis showed quite separate traits for N, E, A, and C scales. O is problematic, with three items which did not show any loadings ≥0.30 on any trait. In total, 49 of the 60 NEO-FFI Items represented unique items relating to specific traits. Five items (N1, E12, O1, O4, O8) did not load on any of the first five components (≥0.30). Seven items did not load on their intended dimension (E6, E7, E10, A1, A2, A7, A10). In conclusion, the oblique rotation was able to make the factor-structure more coherent, due to a reduction of simultaneous item loadings from 18 (orthogonal) to 13 (oblique).

### Differences Between Males and Females T1

Independent-sample *t*-tests (E and O), respectively, Mann-Whitney-*U* tests (N, A, and C) were conducted to reveal differences in personality traits between males and females (Table 8). Males showed significantly lower levels of N *p* ≤ 0.0001, *d* = 0.71), O (*p* ≤ 0.000, *d* = 0.51), and A (*p* ≤ 0.0001, *d* = 0.16). No significant differences were observed for E and C (*p* ≥ 0.05).

**Table 8:** Gender differences in NEO-FFI traits (*n* = 378, raw scores).

**Trait**	**Males (*****n*** **=** **210)**		**Females (*****n*** **=** **168)**				
	**Mean**	**SD**	**Mean**	**SD**	**t/U**	***p***	***d***
N	13.80	6.08	17.84	7.35	11,945	0.000	0.58
E	31.06	4.72	31.07	5.45	−0.016	0.987	0.00
O	23.73	4.59	26.42	5.90	−4.86	0.000	0.52
A	30.67	4.14	34.15	4.87	9,547	0.000	0.86
C	37.04	5.13	36.04	6.32	16,315	0.209	0.13

*Independent-sample t-test for O and A; Mann Whitney U-test for N, A and C; d = effect size (Cohen's d)*.

## Discussion

In settings with limited time access to participants, unable or unwilling compliance for long assessments, short and reliable instruments are necessary to measure relevant information for both research and practice. In the area of sport psychological diagnostics, the NEO-FFI delivers the possibility of rapid screenings to assess the big-five personality traits. In high-level sports and, respectively, soccer, researchers, coaches, and sports psychologists alike require information about quality criteria of the employed instrument such as internal and test-retest reliability, factor structure, and stability in their specific field to avoid over-generalizing or misinterpreting results based on non-comparable populations. As research of the FFM in high-performance athletes is lacking, the present study aimed to fill this gap and focus on the suitability of the NEO-FFI to measure personality traits specifically within elite-level soccer athletes.

Analysis of internal consistency across all athletes showed similar values in comparison to other results outside sport in the English (Caruso, 2000), French (Rolland et al., 1998) and German (Borkenau and Ostendorf, 2008) versions: N and C had the highest Cronbach's alpha levels (0.83 and 0.83, respectively), whereas E, O, and A all shared similar albeit lower alpha levels (0.69, 0.68, and 0.68, respectively). More specifically, females had higher internal consistency outcomes across each trait than males. This is in line with the large scale by Caruso (2000) who combined 51 samples in which the NEO Instruments PI, PI-R and FFI were combined, also reporting that females displayed higher alpha levels for every personality trait. Our finding could be explained by higher preciseness during the answering process in the female group, which we perceived a lot in the assessments. Opposingly, not all research reports gender differences for internal consistencies in the NEO questionnaires. For example, some studies reported reliability measures to be similar across genders (Egan et al., 2000; Borkenau and Ostendorf, 2008). Together, the analogous findings of the components from the NEO-FFI in high performance sporting populations with other general populations supports the transfer and use of the NEO-FFI into professional soccer.

Additionally, results of test-retest reliability are again in a similar range to other studies, although the present study used a different interval between the assessments. Studies with longer intervals like two (Borkenau and Ostendorf, 1993) or four (Robins et al., 2001) years found lower reliabilities, and shorter two-week intervals found comparable results (Robins et al., 2001). A 6-week interval was chosen to reduce impacts of item or answer remembrance (which maybe occur in a 2-week interval) and have a more realistic view of stability and reflect true change with a minimized measurement error (Becker, 2000; Schmidt et al., 2003; Watson, 2004). The findings of the current study show a high level of robustness, without biases of different occasions separated by an interval where no rapid personality changes could be expected. It must also be noted that most of the long interval studies mentioned above are not made with the intention of giving a view into potential applicable instruments in certain setting; rather they focused on longitudinal changes in personality traits. That leads to a lack of information concerning studies with a theoretical background and a specific aim for test-retest data.

In the current study, intercorrelations for the total sample appeared largely in line with similar studies, but with slight differences. For instance, in comparison to Egan et al. (2000) and Borkenau and Ostendorf (2008), the present study reported intercorrelations between all traits except for a positive rather than a negative association between O and C. Furthermore, apart from the correlations between N and E (for both the total and male sample only) and N and C (for female sample only), all correlation coefficients were higher in the present study than what is reported by Borkenau and Ostendorf (2008), whereas only four out of ten coefficients were higher in comparison to Egan et al. (2000).

Even if the non-orthogonality of the factors suggests an oblique rotation, an oblimin rotation was also conducted to determine differences in rotation-methods and have a comparison to previous studies. As expected, the oblique rotation showed better but not exceeding results than the oblimin. Only N and C scales appear to homogenously measure the traits as they should. Yet E, O, and A show more heterogeneity and variance amongst the factors. This is in line with previous findings, where also N and C show the best homogeneity (Egan et al., 2000; Aluja et al., 2005). The current study also replicated the pattern of weak and, respectively, missing loading of items on their intended factor (Egan et al., 2000; McCrae and Costa, 2004; Aluja et al., 2005; Borkenau and Ostendorf, 2008). These aspects of heterogeneous loadings may be attributed to the intercorrelations between the scales and their classification in two higher order factors. Principle component analysis (PCA) confirmed the consensus in literature, that the big five can be assigned to two higher order factors (Digman, 1997; Markon et al., 2005; Chang et al., 2012). PCA of the 60 items and the five-factor solution showed similar explanations of variances as previous studies (Egan et al., 2000; Aluja et al., 2005; Borkenau and Ostendorf, 2008).

## Limitations

A limitation of our study is linked to the misunderstanding of terms. We had several cases where athletes asked for the meanings of different words like “depressed,” “abstract,” “poetry,” or whole statements like “I believe letting students hear controversial speakers can only confuse and mislead them.” The origin of those misunderstanding problems lie in the population, respectively, samples which were chosen for development and evaluation of the NEO-FFI (Bodner, 2006). Those samples mostly exist of well-educated subjects and might not reflect the average type of elite-level soccer athletes. This case leads to difficulties when such questionnaires get applied without an immediately available consultant. As many instruments nowadays are applied using online software that can be answered from everywhere, future instruments should aim to prevent abstract and difficult expressions to understand. A second limitation lies in the biases like social desirability or role thinking when answering these questions. Athletes may often report what they believe is the right answer within a sporting context despite the instrument being a measure of non-sporting specific questions. Such mindsets may alter the way in which they answer the questions, as personality characteristics may be contextually dependent (i.e., different on and off the field). For instance, a team-captain get asked about leadership, and he/she may immediately think about their role in the team, despite them being not highly into leadership from a trait point of view. Additionally, the sample size could be one limitational aspect that influences the divergent factor loadings in our and similar studies with smaller or specific niche samples.

## Conclusion

The current study implemented the NEO-FFI to measure the personality traits of a large sample of high-level soccer athletes and to examine the suitability of the use of NEO-FFI as a measure of the FFM for elite soccer players. The results demonstrated that the NEO-FFI had similar findings for (test-retest-) reliability, factor structure and stability in the elite-level soccer environment as previously reported in various other general populations. This study supports the use of the NEO-FFI as a time-efficient and reliable personality instrument that can inform staff, players, and researchers alike on the unique personality characteristics of each athlete. It would be beneficial for more studies to continue to investigate the NEO-FFI in various other high-performance sports in order to better generalize the findings of this study.

## Data Availability Statement

The datasets presented in this article are not readily available because the dataset includes information about national and international elite-athletes. Therefore, the dataset is under restrictions. Requests to access the datasets should be directed to jan.spielmann@tsg-researchlab.de.

## Ethics Statement

The studies involving human participants were reviewed and approved by Universität des Saarlandes Ethikkommission der Fakultät HW Campus A1 3 66123 Saarbrücken Ethics Approval Number: 19/19. Written informed consent to participate in this study was provided by the participants' legal guardian/next of kin.

## Author Contributions

JS: conceptualization, methodology, formal analysis, investigation, writing—original draft, writing—review & editing, and project administration. AB: writing—original draft and writing—review & editing. JM: conceptualization, resources, and supervision. All authors contributed to the article and approved the submitted version.

## Conflict of Interest

The authors declare that the research was conducted in the absence of any commercial or financial relationships that could be construed as a potential conflict of interest.

## Publisher's Note

All claims expressed in this article are solely those of the authors and do not necessarily represent those of their affiliated organizations, or those of the publisher, the editors and the reviewers. Any product that may be evaluated in this article, or claim that may be made by its manufacturer, is not guaranteed or endorsed by the publisher.
